# Antioxidant Effect of *Stryphnodendron rotundifolium* Martius Extracts from Cariri-Ceará State (Brazil): Potential Involvement in Its Therapeutic Use

**DOI:** 10.3390/molecules17010934

**Published:** 2012-01-18

**Authors:** José Galberto Martins da Costa, Gerlânia de Oliveira Leite, Albys Ferrer Dubois, Rodrigo Lopes Seeger, Aline Augusti Boligon, Margareth Linde Athayde, Adriana Rolim Campos, João Batista Teixeira da Rocha

**Affiliations:** 1 Department of Biological Chemistry, Laboratory of Natural Products, Program of Post-Graduation in Molecular Bioprospection, Regional University of Cariri, Street Cel. Antônio Luiz 1161, Pimenta, 63105-000 Crato-CE, Brazil; 2 National Centre of Applied Electromagnetism, University of Oriente, Avenida de Las Américas, s/n. CP 90900, Santiago de Cuba, Cuba; 3 Program of Post-Graduation in Biological Sciences-Biochemical Toxicology, Federal University of Santa Maria, Campus Camobi, Santa Maria, RS, 97105-900, Brazil; 4 Program of Post-Graduation Pharmaceutical Science, Federal University of Santa Maria, Campus Camobi, Santa Maria, RS, 97105-900, Brazil; 5 Vice-Rectory of Research and Post-Graduation, University of Fortaleza, Av. Washington Soares1321, Edson Queiroz, 60811-905, Fortaleza, CE, Brazil

**Keywords:** *Stryphnodendron rotundifolium*, antioxidant effects, oxidative stress, phenolic compounds, HPLC/DAD

## Abstract

*Stryphnodendron rotundifolium* is a phytotherapic used in the northeast of Brazil for the treatment of inflammatory processes which normally are associated with oxidative stress. Consequently, we have tested the antioxidant properties of hydroalcoholic (HAB) and aqueous extracts (AB) from the bark and aqueous extract (AL) from the leaves of *Stryphnodendron rotundifolium* to determine a possible association between antioxidant activity and the popular use of this plant. Free radical scavenger properties were assessed by the quenching of 1′,1′-diphenil-2-picrylhydrazyl (DPPH) and the calculated IC_50_ were: HAB = 5.4 ± 0.7, AB = 12.0 ± 2.6, and AL = 46.3 ± 12.3 µg/mL. Total phenolic contents were: HAB = 102.7 ± 2.8, AB = 114.4 ± 14.6, and AL = 93.8 ± 9.1 µg/mg plant). HPLC/DAD analyses indicated that gallic acid, catechin, rutin and caffeic acid were the major components of the crude extracts of *S. rotundifolium*. Plant extracts inhibited Fe(II)-induced lipid peroxidation in brain homogenates. Iron chelation was also investigated and only HBA exhibited a weak activity. Taken together, the results suggest that *S. rotundifolium* could be considered an effective agent in the prevention of diseases associated with oxidative stress.

## 1. Introduction

Under normal circumstances, reactive oxygen species (ROS) such as O_2_^•−^, •OH, LOO^•^ and H_2_O_2_ are detoxified by an efficient antioxidant system that includes enzymes such as superoxide dismutase, catalase and glutathione peroxidases. However, an overproduction of reactive species or a decline in the natural antioxidant defenses can produce cellular oxidative stress which can be found in a variety of chronic inflammatory diseases such as arthritis and atherosclerosis. In addition, oxidative stress is thought to be involved in other ailments associated with aging, *viz.* cancer, diabetes, hepatitis, and neurodegeneration [1,2,3]. In fact, literature data have supported a central role for H_2_O_2_ and ROS as important factor governing chronic degenerative diseases and lifespan in living organisms [4,5].

Medicinal plants have been traditionally used in the treatment of several human diseases and their pharmacological and therapeutic properties have been attributed to different chemical constituents isolated from their crude extracts. Of particular importance, chemical constituents with antioxidant activity can be found at high concentrations in a variety of plants and can be responsible for their preventive effects in various degenerative diseases, including cancer, neurologic, and cardiovascular diseases [6,7,8].

*Stryphnodendron rotundifolium* Martius, popularly known as “barbatimão”, is a typical tree of the Cariri region, Ceará State, Brazil. Phytochemical analysis of the ethanol extract of the bark of *S. rotundifolium* allowed the detection of tannins, flavonoids, and alkaloids [9,10]. Several species of this Genus have been used in folk medicine for wound healing, leucorrhea, gynecological problems, bactericidal, antihypertensive, anti-ulcerogenic and as anti-inflammatory agent [11,12,13,14,15,16,17,18,19], which at least in part can be associated with bark’s high content of tannins [11,13,20,21]. It is important to comment about the congener species *S. adstringens* (Mart.) Coville, which was described in the 1st edition of Brazilian Pharmacopoeia (1929), traditionally used as astringent due to the high tannin content (20~30%) of its bark [11,22] and the most studied species, given that its it believed to be the “true barbatimão”.

In this context, considering the importance of the oxidative stress in the pathogenesis of various diseases, including those related to the Central Nervous System and the presence of a number of compounds with antioxidant properties in the plant extracts, the aim of the present study was to investigate the antioxidant activity of different crude extracts of *S. rotundifolium*, a plant popularly used in folk medicine in Ceará State. 

## 2. Results

### 2.1. Total Phenolic Content and HPLC/DAD Analyses

**Figure 1:**
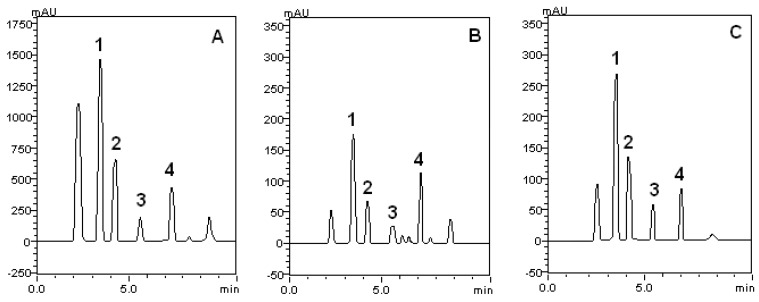
High performance liquid chromatography profile of *S. rotundifolium*: (**A**): hydroalcoholic bark extract; (**B**): aqueous leaves extract; (**C**): aqueous bark extract. Gallic acid (peak 1), catechin (peak 2), caffeic acid (peak 3) and rutin (peak 4).

Total phenolic contents of aqueous extract from leaves (AL) (93.8 ± 9.1 µg of phenol/mg plant, n = 9), aqueous extract from bark (AB) (114.4 ± 14.6, n = 7), and hydroalcoholic extract from bark (HAB) (102.7 ± 2.8, n = 5) were not statistically different, although a tiny predominance of aqueous bark phenolic contents could be seen. Considering the bark extraction procedures, no significant difference in total phenolic content could be detected between aqueous extractions alone in respect to its mixture with alcohol in equal volume (1:1). The chemical composition of extracts was investigated using HPLC. A representative chromatogram of each extract is shown in Figure 1 and the quantification of major components identified in the extracts are depicted in Table 1. Gallic acid was the major component of the extracts and it was more concentrated in bark, especially in the hydroalcoholic extract, than in leaves extracts (Table 1). Rutin, caffeic acid and catechin were also detected in the extracts and their contents were in the order:

hydroalcoholic bark > aqueous bark ≥ aqueous leaves

**Table 1:** Phenolics and flavonoids composition of *S. rotundifolium*.

Compounds	Hydroalcoholic bark		Aqueous leaves		Aqueous bark	
	mg/g	%	mg/g	%	mg/g	%
Gallic acid	210.8 ± 0.27	21.08	56.5 ± 0.09	5.65	89.3 ± 0.10	8.93
Catechin	160.4 ± 0.06	16.04	11.9 ± 0.27	1.19	50.7 ± 0.32	5.07
Caffeic acid	51.5 ± 0.18	5.15	3.2 ± 0.16	0.32	4.8 ± 0.05	0.48
Rutin	102.5 ± 0.34	10.25	31.2 ± 0.04	3.12	20.9 ± 0.17	2.09

Results are expressed as mean ± SEM performed in triplicate.

### 2.2. Effects of *S. rotundifolium* on Iron Sulfate-Induced TBARS Production

**Figure 2:**
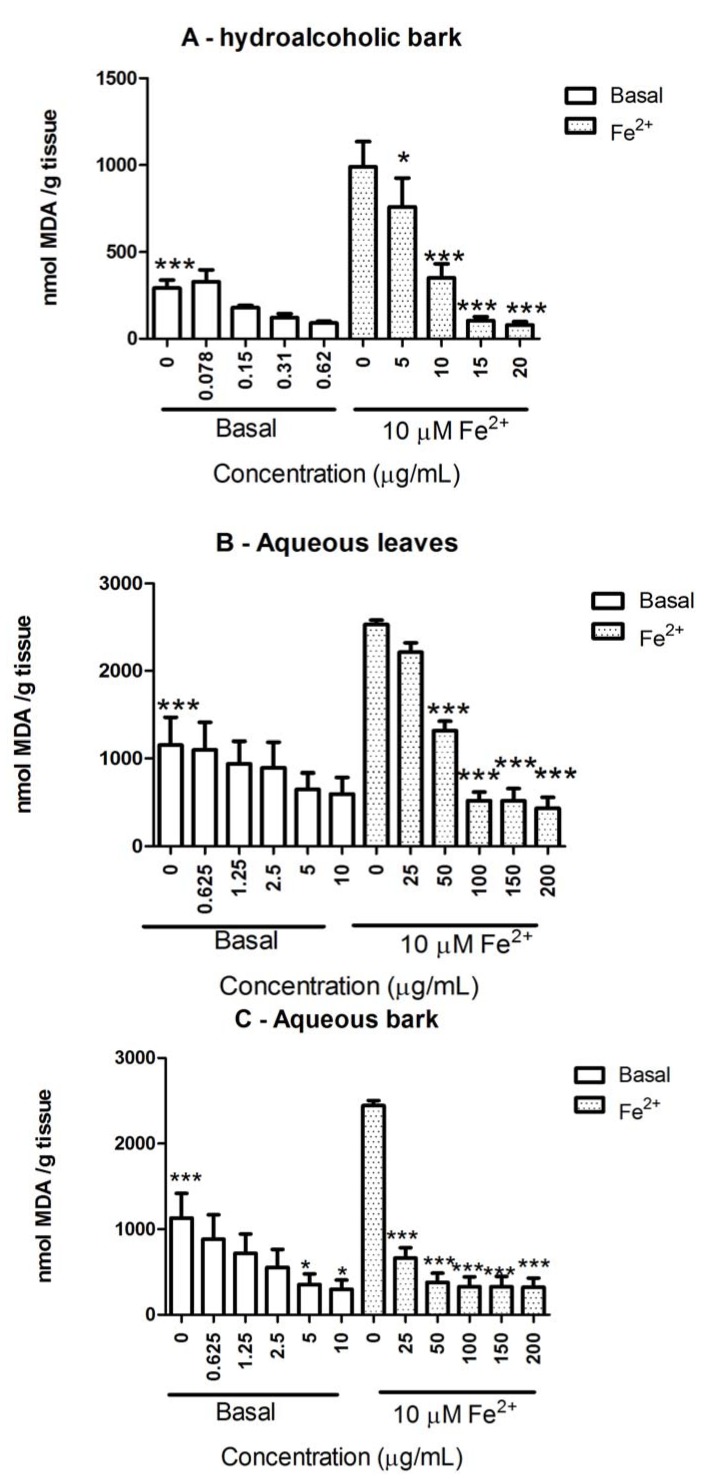
Antioxidant properties of different extracts from *S. rotundifolium* Mart.: (**A**): hydroalcoholic bark extract; (**B**): aqueous leaves extract; (**C**): aqueous bark extract. Lipid peroxidation (TBARS production) in brain homogenates was determined either in the absence or in the presence of Fe^2+^ (10 µM). Values are expressed as mean ± SEM from 3 to 4 independent experiments performed in duplicate. *** *p* < 0.001 *vs*. Fe^2+^-induced TBARS; * *p* < 0.05 *vs*. Fe^2+^-induced TBARS; *** *p* < 0.001 *vs*. basal.

Fe^2+^ (10 µM) induced a significant stimulation of brain TBARS levels (*p* < 0.05), which were partially reduced by *S. rotundifolium* extracts in a concentration-dependent manner (*p* < 0.05; Figure 2). The antioxidant potency of the plant varied depending on the extract tested and, generally, the extracts were more potent against basal than against iron-stimulated TBARS production (Figure 2).

For basal TBARS levels, the potency of the extracts was in the following order:

hydroalcoholic bark > aqueous bark = aqueous leaves

For iron-induced TBARS production, the order was:

hydroalcoholic bark (HAB) > aqueous bark (AB) > aqueous leaves (AL)

The IC_50_ values for TBARS, for hydroalcoholic bark (HAB), aqueous bark (AB), and aqueous leaves (AL) extracts from *S. rotundifolium* are show in Table 2.

**Table 2:** IC_50_ values (µg/mL) for TBARS, DPPH, iron chelation and deoxyribose degradation by hydroalcoholic bark (HAB), aqueous bark (AB), and aqueous leaves (AL) extracts from *S. rotundifolium*.

	TBARS		DPPH	Iron chelation	Deoxyribosedegradation
	Basal	Fe^2+^			
HAB	0.24 ± 0.02	7.00 ± 1.04 ***	5.43 ± 0.73 ***	>200	>100
AB	1.97 ± 0.44	13.58 ± 2.00 ***	12.00 ± 2.67 ***	>200	>100
AL	4.58 ± 1.73	60.00 ± 7.64	46.33 ± 12.35	>200	>100

*** *p* < 0.001 *vs*. aqueous leaves extracts from *S. rotundifolium* (Fe^2+^-induced TBARS and DPPH).

### 2.3. Deoxyribose Degradation

**Figure 3:**
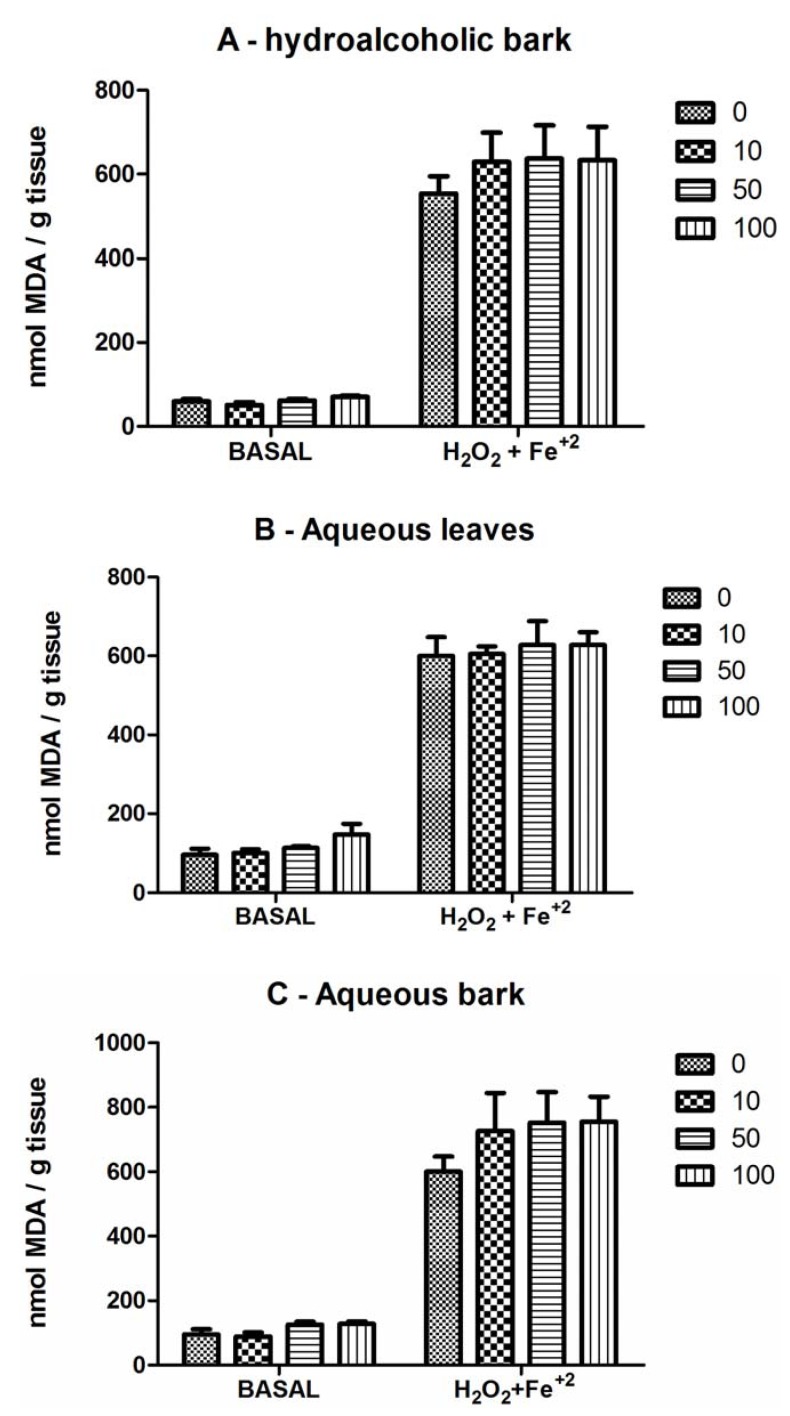
S. rotundifolium extracts did not inhibit Fenton’s reaction. (**A**) hydroalcoholic bark extract; (**B**) aqueous leaves extract; (**C**) aqueous bark extract on basal and Fe^2+^ (10 µM) + H_2_O_2_ (1 mM)-induced deoxyribose degradation. Deoxyribose was incubated for 20 min with or without H_2_O_2_ or Fe^2+^ + H_2_O_2_ in the presence or absence of extracts. Data are mean ± SEM. Values average from 3 to 4 independent experiments performed in duplicate.

Deoxyribose degradation was stimulated by Fe^2+^ plus H_2_O_2_ and *S. rotundifolium* extracts did not reduce deoxyribose induced by Fenton’s reaction (Figure 3).

### 2.4. DPPH

**Figure 4:**
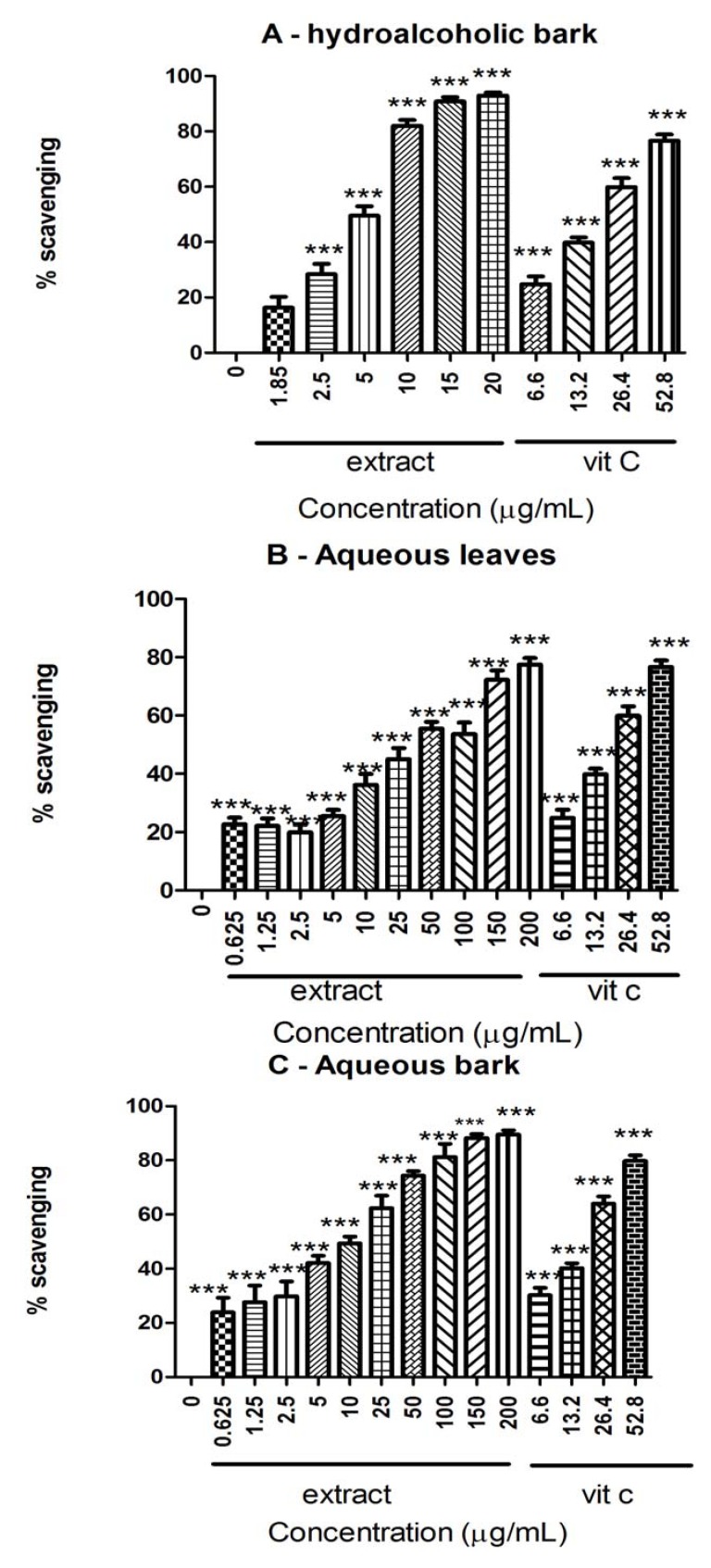
DPPH radical scavenging activity by extracts from *S. rotundifolium*: (**A**) hydroalcoholic extract of bark; (**B**) aqueous extract of leaves; (**C**) aqueous extract of bark. The results are expressed as percentage of inhibition and ascorbic acid was used as a positive control. Data show means ± SEM values average from 3 to 4 independent experiments performed in triplicate. *** *p* < 0.001 *vs*. Control.

*S. rotundifolium* extracts scavenged DPPH radical in a concentration-dependent manner (*p* < 0.05, Figure 4). The antioxidant potency varied depending on the extract tested and it was in the following order for the IC_50_ calculated values: hydroalcoholic bark (HAB) > aqueous bark (AB) > aqueous leaves (AL) (Table 2).

### 2.5. Iron Chelation Assay

**Figure 5:**
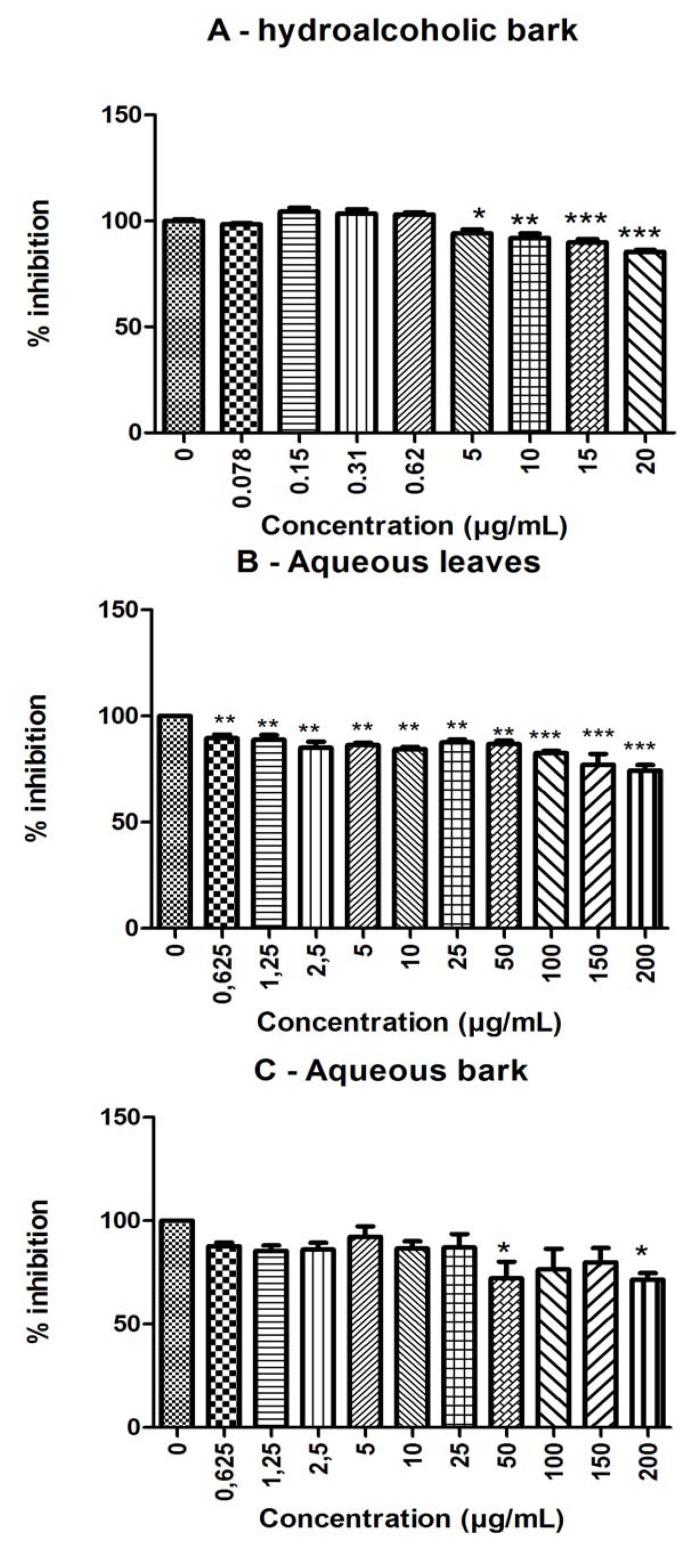
Effects of different extracts from *S. rotundifolium* on iron chelation: (**A**) hydroalcoholic bark; (**B**) aqueous leaves; (**C**) aqueous bark. Data show means ± SEM values average from 3 to 4 independent experiments performed in triplicate. *** *p* < 0.001 *vs*. Control; ** *p* < 0.01 *vs*. Control; * p < 0.05 *vs*. Control.

*S. rotundifolium* extracts exhibited weak iron chelation properties (Figure 5 and Table 2). In fact, the hydroalcoholic bark (HAB) extract caused a maximal chelation (*i.e.*, a decrease in the color intensity of iron-*ortho*-phenantroline complex) of about 20%. A similar result was obtained using leaves aqueous extract. Aqueous bark extract was the less effective as a chelator of iron and caused a significant decrease in the color intensity of iron-*ortho*-phenantroline complex only at highest concentration tested.

## 3. Discussion

In spite of the great cultural and biological diversity found in Cariri (“Chapada do Araripe”), few ethnobothanical and pharmacological studies have been undertaken in this region. Of particular importance, many plant species are widely used in folk medicine for wound healing and treatment of leucorrhea and diarrheoea, including the bark of several species of the genus *Stryphnodendron*. However, there are only few studies regarding *S*. *rotundifolium* and it has been demonstrated that the ethanolic extract from the stem bark extract exhibited antimicrobial and antiulcer activity [9,10]. Literature data have indicated that antioxidant components from exogenous sources, especially from plants and foods, can’ help cell in counteracting oxidative stress [23,24] and several studies have focused in the use of natural therapeutic antioxidant compounds that can afford protection in a variety of *in vitro* and *in vivo* models of human pathologies, including neurotoxicity models [25,26,27,28,29,30]. These antioxidants can be abundantly found in plants in the form of phenolic compounds (flavonoids, phenolic acids and alcohols, stilbenes, tocopherol, tocotrienols) ascorbic acid, carotenoids, and tannins [31,32]. The potential beneficial effect of plants used sporadically for the treatment of inflammatory diseases could be in part linked to their antioxidant activity. Accordingly, inflammation is associated with oxidative stress and a negative modulation of ROS could ameliorate the toxicity of an exacerbated inflammatory response [32].

In this study, we have tested the effect of three extracts from *S. rotundifolium* against iron, a well-known pro-oxidant, to investigate the potential role of its antioxidant constituents on the therapeutic effect of *S. rotundifolium* against pathologies associated with inflammation. Accordingly, *S. rotundifolium* extracts have appreciable amounts of phenolic compounds, including gallic acid and catechin, caffeic acid and rutin. Phenolic compounds are one of the largest groups of metabolites and there are a great interest in their antioxidant, anti-inflammatory, antimutagenic and anticarcinogenic activity [33,34,35,36]. Results presented here clearly indicated that the extracts from *S. rotundifolium* exhibited antioxidant activity in different *in vitro* models. Of potential pharmacological importance, the results have indicated that *S. rotundifolium* extracts, particularly the hydroalcoholic extract from the bark, prevented oxidative damage in brain preparations induced by iron. Free iron can induce neurotoxicity via stimulation of Fenton reaction and its levels can be increased in some degenerative diseases. In fact, *S. rotundifolium* extracts blocked basal and iron-induced TBARS production, though with a higher potency against basal than against iron-induced lipid peroxidation. However, the extracts did not block Fenton’s reaction (as determined by deoxyribose degradation) and had only a weak Fe^2+^ chelating property at relatively high concentrations.

Furthermore, *S. rotundifolium* extracts scavenged DPPH, evidencing their anti-radical properties. DPPH is a stable free radical that accepts an electron or hydrogen radical to become a stable diamagnetic molecule. The scavenging of DPPH radical is widely used for rapid evaluation of antioxidant activity of different compounds [37], and it is well established the inhibitory effect of tannins and flavonoids against DPPH radical [29,30,37,38]. The IC_50_ obtained here were comparable with those obtained by Zocoler [35], working with *S. obovatum* extracts. In fact, the antioxidant potency was associated with polyphenol content. Similarly, here we observed that hydroalcoholic extract from the bark (HAB, IC_50_ = 5.43 ± 0.73 µg/mL) of *S. rotundifolium* presented the lowest IC_50_ value and the highest values of individual phenolics, especially gallic acid, when compared with the other two extracts (AB and AL; Table 1). HPLC analysis indicated the presence of appreciable, but different quantities of gallic acid, catechin, rutin, and caffeic acid in the extracts obtained from *S. rotundifolium* and they are very effective antioxidants [33,39]. These major components can be responsible for the antioxidant properties of crude extracts from *S. rotundifolium*. In fact, gallic acid and catechin contributed with about 40% of tannins related compounds found in these extracts (Table 1). Of particular importance, the high antioxidant properties of these isolated compounds obtained from different plant species [25,33,36,40] can explain the antioxidant activity of crude extracts of *S. rotundifolium*. It is important to remark that this is the first report indicating the presence of high quantities of gallic acid, catechin, rutin and caffeic acid in *S. rotundifolium*. 

## 4. Experimental

### 4.1. Plant Material and Extracts Preparation

Leaves and bark of *S. rotundifolium* was collected in line D of the National Forest Araripe, Crato, Ceará State, Brazil and identified by Professor Afrânio Gomes Fernandes (Universidade Federal do Ceará, Fortaleza). A voucher specimen (#33621) is deposited at the Herbarium Prisco Bezerra, Universidade Federal do Ceará. The aqueous extracts from leaves and barks were obtained by infusion in hot water in a proportion of 3.5% (w/v) and they were prepared just before use. For the hydroalcoholic extract, barks were washed with running water, dried and milled. The resulting powder (420 g) was mixed with a solution of ethanol-water (1:1 v/v, 1.5 L) and maintained for three days at room temperature. The extractive solution was filtered and concentrated under low-pressure to remove ethanol. Then, the resulting aqueous solution was frozen and lyophilized, yielding 34.9 g (8.3%) of dry matter. The obtained extracts were: Hydroalcoholic from the bark (HAB), aqueous from the bark (AB) and aqueous from the leaves (AL).

### 4.2. Chemicals

Tris-HCl, thiobarbituric acid (TBA), 1′-1′-diphenyl-2′-pycrylhydrazyl (DPPH), catechin, gallic acid and malonaldehyde bis-(dimethyl acetal) (MDA) were obtained from Sigma (St. Louis, MO, USA). Iron sulfate (FeSO_4_), caffeic and ascorbic acids, rutin, chloridric and acetic acids were obtained from Merck (Rio de Janeiro, Brazil), CH_3_CN and MeOH (HPLC grade) were from Merck (Darmstadt, Germany), 85% formic acid was provided by Carlo Erba, (Milan, Italy). Water was purified by a Milli-Qplus system from Millipore (Milford, MA, USA). Membrane filter (PRFE 0.45 mm) was purchased from Waters Co. (Milford, MA, USA). All laboratory chemicals used in this study were of reagent grade.

### 4.3. Animals

Male Wistar rats (3.5–3.9 months of age and weighing 270–320 g) were maintained in groups of 3–4 rats per cage. They had continuous access to food and water in a room with controlled temperature (22 ± 3 °C) and on a 12-h light/dark cycle with lights on at 7:00 a.m. The animals were maintained and used in accordance to the guidelines of the Brazilian Association for Laboratory Animal Science (COBEA, register number in the ethic committee 42/2010 of UFSM).

### 4.4. Tissue Preparation

Rats were killed and the encephalic tissue was rapidly dissected and placed on ice. Tissues were immediately homogenized in cold 100 mM Tris-HCl, pH 7.5 (1/10, w/v). The homogenate was centrifuged for 10 min at 4,000× *g* to yield a pellet that was discarded and a low-speed supernatant (S1) that was used for the TBARS assay [41].

### 4.5. TBARS

Production of TBARS was determined using a modified method of Ohkawa [42]. The brain homogenates (100 µL) were incubated at 37 °C for 1 h with or without freshly prepared oxidant (iron sulfate, 50 µL) and different concentrations of the plant extracts together with an appropriate volume of deionized water to give a total volume of 300 µL. The color reaction was carried out by adding 200, 500, and 500 µL of the 8.1% sodium dodecyl sulfate (SDS), acetic acid (pH 3.4) and 0.6% TBA, respectively. The reaction mixtures, including those of serial dilutions of 0.03 mM standard MDA, were incubated at 97 °C for 1 h. The absorbance was read after cooling the tubes at a wavelength of 532 nm in a spectrophotometer. TBARS unit was expressed as nmol/g tissue.

### 4.6. Deoxyribose Degradation

Deoxyribose degradation was determined by method of Halliwell [43]. Deoxyribose is degraded by hydroxyl radicals with the release of thiobarbituric acid (TBA) reactive substances. Deoxyribose (3 mM) was incubated at 37 °C for 30 min with 50 mM potassium phosphate (pH 7.5) plus 0.1 mM iron sulfate (FeSO_4_) and/or 1 mM H_2_O_2_ to induce deoxyribose degradation and extract. After incubation, TBA 0.8% (0.4 mL) and TCA 2.8% (0.8 mL) were added, and the tubes were heated for 20 min at 100 °C and absorbance determined at 532 nm.

### 4.7. DPPH Radical Scavenging

Scavenging of the stable DPPH radical (ethanolic solution of 0.25 mM) was assayed *in vitro* [44]. The mixture was shaken and allowed to stand at room temperature for 30 min and the absorbance was measured at 517 nm in a spectrophotometer. Percentage inhibition was calculated from the control (ascorbic acid).

### 4.8. Iron Chelation Assay

The ability of extracts to chelate Fe(II) was determined using the modified method described by Puntel [45]. Briefly, freshly prepared 7.5 mM FeSO_4_ (20 µL) was added to a reaction mixture containing 0.1 M Tris-HCl (pH 7.4, 168 µL), saline (218 µL) and the aqueous extract of the plant. The reaction mixture was incubated for 5 min, before the addition of 0.25% 1,1-*O*-phenanthroline (w/v, 13 µL). The absorbance was subsequently measured at 510 nm in a spectrophotometer.

### 4.9. Phenolics Content

The total phenol content was determined by adding the extracts (50 µL) to 10% Folin-Ciocalteau’s reagent (v/v, 200 µL), 7.5% sodium carbonate (400 µL) and water (1,350 µL). The reaction mixture was incubated at 45 °C for 40 min, and the absorbance was measured at 765 nm in a spectrophotometer. Gallic acid was used as a standard phenol [46]. The mean of three readings was used and the total phenol content was expressed as milligrams of gallic acid equivalents/g extract.

### 4.10. HPLC/DAD Analysis

High performance liquid chromatography was performed with a Prominence Auto Sampler (SIL 20A) HPLC system (Shimadzu, Kyoto, Japan), equipped with Shimadzu LC-20AT reciprocating pumps connected to a DGU 20A5 degasser, CBM 20A integrator, SPD-M20A diode array (DAD) UV-VIS detector and Software LC solution 1.22SP1. Chromatographic analyses were carried out in isocratic conditions using a RP-C18 column (4.6 mm × 250 mm) packed with 5 µm diameter particles. The mobile phase was methanol-acetonitrile-water (40:10:45, v/v/v) containing 1.0% acetic acid [25]. The flow rate was 0.8 mL/min, injection volume 50 µL and the wavelength were 257 nm for gallic acid, 280 nm for catechin, 325 nm for caffeic acid and 365 nm for rutin. The mobile phase was filtered through a membrane filter 0.45 µm and then degassed in an ultrasonic bath before use. The standard solutions of rutin, catechin, caffeic and gallic acids were prepared in the same mobile phase of HPLC analysis. Standard calibration curves were prepared in a concentration range of 0.0125 to 0.200 mg/mL. Chromatographic peaks were confirmed by comparing its retention time with those of reference standards and by DAD spectra (200 to 500 nm). Quantification was performed by peak integration using the external standard method. The calibration curve for caffeic acid was: Y = 12153X − 21513, r = 0.9983, gallic acid: Y = 10913X − 52631, r = 0.9998, catechin: Y = 11355X − 10471, r = 0.9968 and rutin: Y = 19217X − 16949, r = 1. All chromatographic operations were performed at room temperature and in triplicate.

### 4.11. Statistical Analysis

Data from TBARS and iron chelation were analyzed by on-way ANOVA, followed by Student-Newman-Keuls multiple comparison when appropriated. Calibration curves and correlation coefficients (r) were constructed and calculated by linear regression using MS Excel for Windows.

## 5. Conclusions

In conclusion, crude extracts from *S. rotundifolium* exhibited important *in vitro* antioxidant properties (inhibition of TBARS production and DPPH scavenging activity). In part, these effects can be associated with the presence of high amounts of gallic acid and other polyphenolic compounds in *S. rotundifolium* crude extracts. Consequently, the antioxidant activity of *S. rotundifolium* could be involved at least in part in the therapeutic use of *S. rotundifolium* and this plant should be considered as a potential alternative source of pharmacological active principles for the prevention of various neurological diseases associated with oxidative damage, confirming the ethnopharmacological value of the genus.
